# Supplementary material for: Higher Level of Sports Activities Participation during Five-Year Survival Is Associated with Better Quality of Life among Chinese Breast Cancer Survivors
Source: Cancers (Basel). 2021 Dec 1;13(23):6056. doi: 10.3390/cancers13236056 (PMC8656618; doi:10.3390/cancers13236056)
Supplement: Supplementary file 1 [file cancers-13-06056-s001.zip › cancers-1430800-supplementary.pdf]

Supplementary Table S1. Modified Chinese Baecke Questionnaire [25]

**Work Index**		
1. What is your main occupation?…		1-3-5
2. Do you often sit at work?	Never/seldom/sometimes/always	4 - 3 - 2 - 1
3. Do you often stand at work?	Never/seldom/sometimes/always	1 - 2 - 3 - 4
4. Do you often walk at work?	Never/seldom/sometimes/always	1 - 2 - 3 - 4
5. Do you often lift heavy loads at work?	Never/seldom/sometimes/always	1 - 2 - 3 - 4
6. Do you often feel physically tired after working?	Never/seldom/sometimes/always	1 - 2 - 3 - 4
7. Do you often sweat at work?	Never/seldom/sometimes/always	1 - 2 - 3 - 4
8. When compared with people of your own age and sex, do you think your work is physically much heavier or lighter?	Lighter/as heavy/heavier	1 - 2 - 3
**Sport Index**		
9. Do you play sport when you don't need to work or do household activities? Yes/No<br>If yes: –which sport do you play most frequently? . . .<br>–how many hours a week?<br>–how many months a year?<br>If you play a second sport:<br>–which sport is it? . . .<br>–how many hours a week?<br>–how many months a year?	 <br>1 / 3 / 5<br>0/<1/1-2/2-3/3-4/>4<br>0/<1/1-3/4-6/7-9/>9<br><br>1 / 3 / 5<br>0/<1/1-2/2-3/3-4/>4<br>0/<1/1-3/4-6/7-9/>9	 <br>0.76-1.26-1.76<br>0-0.5-1.5-2.5-3.5-4.5<br>0-0.04-0.17-0.42-0.67-0.92<br><br>0.76-1.26-1.76<br>0-0.5-1.5-2.5-3.5-4.5<br>0-0.04-0.17-0.42-0.67-0.92
10. In comparison with others of my own age I think my physical activity during leisure time is . . .	Less/the same/more	1 - 2 - 3
11. Do you play sport during leisure time?	Never/seldom/sometimes/often	1 - 2 - 3 - 4
**Leisure Time Index**		
12. Do you often watch television/video/laser disc/see movie during leisure time?	Never/seldom/sometimes/always	4 - 3 - 2 - 1
13. Do you often walk for pleasure during leisure time?	Never/seldom/sometimes/always	1 - 2 - 3 - 4
14. How many minutes per day do you walk and/or cycle to and from work, school, and shopping?	<10/10-30/30-60/60-120/>120	1 - 2 - 3 - 4 - 5
15. Do you often go shopping (not including grocery shopping) during leisure time?	Never/seldom/sometimes/often/very often	1 - 2 - 3 - 4
16. Do you often play mahjong during leisure time?	Never/seldom/sometimes/often/very often	4 - 3 - 2 - 1
**Housework Index**		
17. Do you often do heavy household work (e.g., extensive clean-up, washing floors, washing windows, washing toilet, washing kitchen, cleaning all furniture and electrical appliances, hanging wet linens, etc.)?	Never/seldom/sometimes/often	1 - 2 - 3 - 4

18. Do you often do light household work (e.g., general clean-up, dusting, vacuuming, sweeping floor, handwashing clothes, hanging clothes, ironing, hanging linen, etc.)?	Never/seldom/sometimes/often	1 - 2 - 3 - 4
19. Do you often prepare meals?	Never/seldom/sometimes/often	1 - 2 - 3 - 4
20. Do you often wash dishes?	Never/seldom/sometimes/often	1 - 2 - 3 - 4
21. Do you often do grocery shopping?	Never/seldom/sometimes/often	1 - 2 - 3 - 4
22. How many days per week do you go to the market?	1-2/3-4/5-7	1 - 2 - 3
23. How many minutes/hours per day on average do you walk to and from grocery shopping (not including transportation time)?<br>(If not doing grocery shopping every day, write down on average how many days per week he/she does it.)	<10/10-30/30-60/60-120/>120	1-2-3-4-5
24. How many hours per day do you sleep on average?	<7/7+ hours	1 - 1.5
Calculation of the score for question 9<br>If type of sport = 1, then Intensity = 0.76<br>If type of sport = 3, then Intensity = 1.26<br>If type of sport = 5, then Intensity = 1.76<br><br>If number of hours a week = <1 then Time = 0.5<br>If number of hours a week = 1-2 then Time = 1.5<br>If number of hours a week = 2-3 then Time = 2.5<br>If number of hours a week = 3-4 then Time = 3.5<br>If number of hours a week = >4 then Time = 4.5<br><br>If number of months a year = <1 then Proportion = 0.04<br>If number of months a year = 1-3 then Proportion = 0.17<br>If number of months a year = 4-6 then Proportion = 0.42<br>If number of months a year = 7-9 then Proportion = 0.67<br>If number of months a year = >9 then Proportion = 0.92<br><br>Question 9 = $(Intensity_1 * Time_1 * Proportion_1)$<br>$+ (Intensity_2 * Time_2 * Proportion_2)$	0/0.01-<4/4-<8/8-<12/≥12*<br>*0 is given to people who do not play sport.	1 - 2 - 3 - 4 - 5
Calculation of physical activity indices<br>The subscript for I corresponds to the question item number.<br>WORK INDEX (WI) = $(I_1 + I_2 + I_3 + I_4 + I_5 + I_6 + I_7 + I_8)/8$<br>SPORTS INDEX (SI) = $(I_9 + I_{10} + I_{11})/3$		

LEISURE TIME INDEX (LTI) = $(I_{12} + I_{13} + I_{14} + I_{15} + I_{16})/5$
HOUSEWORK INDEX (HWI) = $(I_{17}+I_{18}+I_{19}+I_{20}+I_{21}+I_{22}+I_{23})/7$
TOTAL INDEX (TI) = = WI + SI + LTI + HWI

Reference 25. Ho SC, Yu R, Chan S: Comparison of the modified Chinese Baecke Questionnaire with a 3-day activity diary in a Hong Kong Chinese population. Asia Pac J Public Health 27:Np2358-71, 2015
